# Endoscopic resection versus surgery for early gastric cancer and precancerous lesions: a meta-analysis

**DOI:** 10.1186/s40064-016-2273-7

**Published:** 2016-05-21

**Authors:** Shulei Zhao, Xiaohua Zhang, Jing Wang, Jian Ge, Jin Liu

**Affiliations:** Department of Gastroenterology, Shandong Provincial Hospital Affiliated to Shandong University, 324 Jingwu Weiqi Rd, Jinan, 250021 China; Department of Hepatology, Shandong Provincial Hospital Affiliated to Shandong University, 324 Jingwu Weiqi Rd, Jinan, 250021 China

**Keywords:** Endoscopic resection, Surgery, Early gastric cancer, Precancerous lesions

## Abstract

**Aim:**

To compare the efficacy and safety of endoscopic resection (ER) and surgery for the treatment of early gastric cancer and precancerous lesions.

**Methods:**

Databases, such as PubMed, EMBASE, Cochrane Library, and Science Citation Index, from 2000 to 2016, were searched for eligible articles. In this meta-analysis, the main outcome measurements were local recurrence, complications, metachronous lesions, hospital stay, and 5-year overall survival.

**Results:**

Nine trials were identified and a total of 2748 patients were included. The rate of complication was higher in the surgery group compared with the ER group (OR 0.41; 95 % CI 0.30–0.55). The rates of local recurrence and metachronous lesions were lower in the surgery group (OR 0.03; 95 % CI 0.00–0.06; OR 8.76; 95 % CI 4.17–18.41). The hospital stay was shorter in the ER group (mean difference −6.96; 95 % CI −7.94 to −5.99). The 5-year overall survival rate did not significantly differ between the two groups (OR 1.23; 95 % CI 1.03–1.47).

**Conclusions:**

We provided evidence that, ER was comparable to surgery in terms of the 5-year overall survival. In addition, ER had a lower rate of complications and shorter hospital stay, but a higher rate of local recurrence and metachronous lesions for the treatment of early gastric cancer and precancerous lesions.

## Background

Gastric cancer is the second most common cause of cancer-related death in the world, and it remains difficult to cure, primarily because most patients present with advanced disease. Early gastric cancer is defined as gastric carcinoma confined to the mucosa and submucosa of the stomach, with or without regional lymph node metastasis (Japanese Gastric Cancer Association 1998, 2011).

Gastrectomy with lymph node dissection remains the standard therapy for early gastric cancer. A variety of complications can develop after surgery, and the patient’s quality of life can be greatly impacted because of the modification of the stomach (Okamura et al. 1988).

Endoscopic resection (ER) methods, such as endoscopic mucosal resection (EMR) and endoscopic submucosal dissection (ESD), are widely accepted for the treatment of superficial gastric cancer without lymph node metastasis, because of the minimal invasion, low cost, good patient tolerance, and better quality of life after the operation, with low adverse event rates (El-Sedfy et al. 2014; Shen et al. 2013).

There is no current consensus on the optimal method for the treatment of early gastric cancer and precancerous lesions. We conducted a systematic literature review to compare the efficacy and safety of ER and surgical methods for the treatment of early gastric cancer and precancerous lesions.

## Methods

### Data sources and searches

We searched databases including PubMed, EMBASE, the Cochrane Library, and Science Citation Index from January 2000 to March 2016 to identify related articles, without language restriction, which compared ER and surgery. All bibliographies were indentified in the reference lists. The search terms were “gastric cancer or gastric neoplasia” and “endoscopic mucosal resection or endoscopic submucosal dissection”. Major proceedings of international meetings (such as Digestive Disease Week and Asian Pacific Digestive Week) were also hand-searched.

### Study selection

The inclusion and exclusion criteria are shown in Table 1.

**Table 1:** The inclusion and exclusion criteria of the study

Inclusion criteria	Exclusion criteria
Early gastric cancer diagnosis for every patient was confirmed by histology	Case report
Comparison of ER and surgical treatment for early gastric cancer	Comment
	Review
	Letter to editor
	Insufficient data
	Guidelines

### Data extraction and quality assessment

Data were extracted by one investigator and confirmed by the other according to a predefined data extraction form. Disagreements were resolved by consultation with a third investigator. The following data were collected: year of publication, first author, country, duration, age, sex, depth of invasion, differentiation and follow up period, the local recurrence rate, procedure-related complications, metachronous lesions, hospital stay, and 5-year overall survival. The Newcastle-Ottawa Scale was used to assess the quality of the included non-randomized studies (Wells et al. 2015).

### Statistical analysis

All extracted data were entered in the freeware program Review Manager (Version 5.0 for Windows, Cochrane Collaboration). The weighted mean difference was recommended for continuous data, and the odds ratio (OR) with 95 % confidence intervals (CI) was recommended for dichotomous data. Statistical heterogeneity between trials was evaluated by the Chi square test and was considered to be present when *P* was less than 0.1. We also used I^2^ to assess the heterogeneity. An I^2^ of more than 50 % was considered to be statistically significant. In the presence of statistical heterogeneity, heterogeneity was explored by subgroup analysis or a random-effects model. Publication bias was detected by a funnel plot, and then the symmetry of the funnel plot was confirmed by the Egger’s test, with a *P* value of 0.05.

## Results

### Study selection

**Fig. 1:**
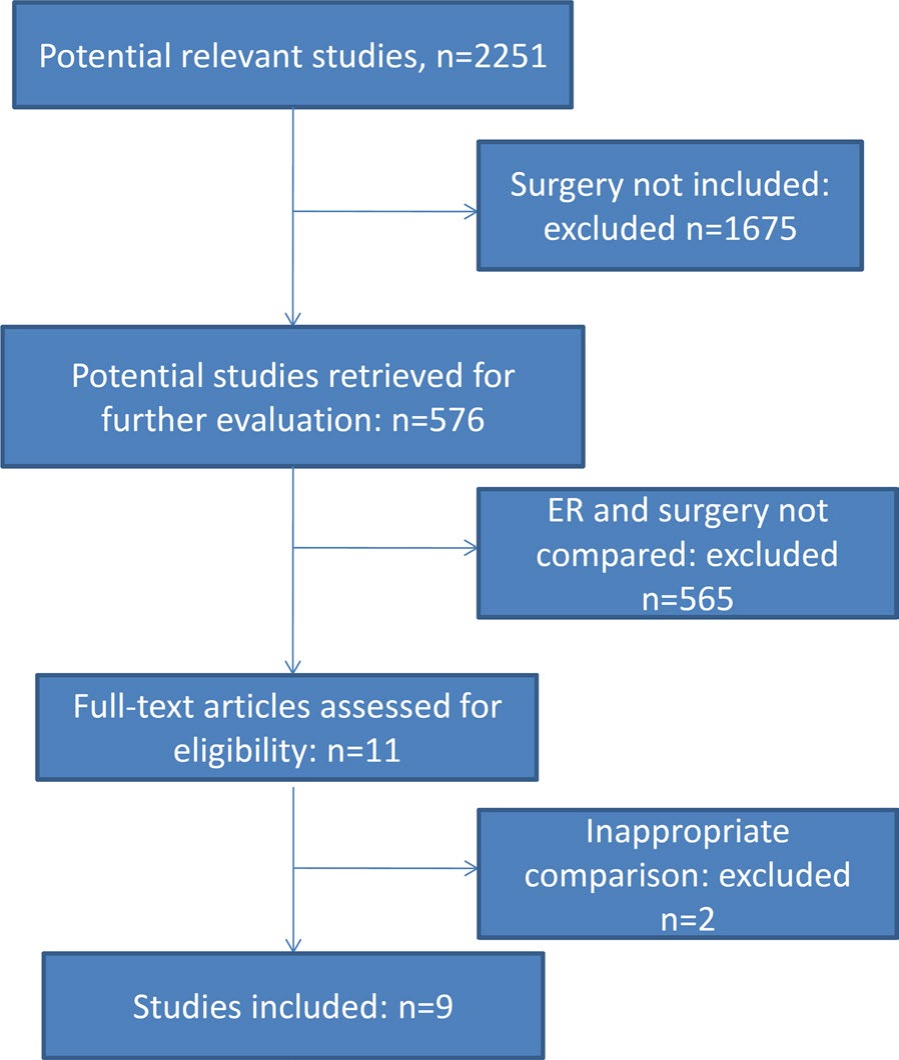
Flow diagram of trial selection

A total of 2251 potential studies were retrieved for the meta-analysis. 1675 were excluded for not including the surgical treatment and 565 were excluded because ER and surgery were not compared. Of the 11 remaining articles, 2 were excluded because they had not compared the main outcomes. The remaining 9 eligible studies (Cho et al. 2015; Song et al. 2015; Choi et al. 2011, 2015; Kim et al. 2014, 2015; Park et al. 2014; Chung et al. 2014; Chiu et al. 2012) were chosen for further analysis (Fig. 1). A total of 2748 patients were included in the meta-analysis, including 1339 patients in the ER group and 1409 patients in the surgery group. Of these studies, 6 included only patients with ESD (Cho et al. 2015; Song et al. 2015; Park et al. 2014; Kim et al. 2014; Chung et al. 2014; Chiu et al. 2012), 1 included only patients with EMR (Choi et al. 2011), and 2 included patients with both ESD and EMR (Choi et al. 2015; Kim et al. 2015). Open surgery or laparoscopic surgery were mentioned in 5 studies (Choi et al. 2015; Kim et al. 2014, 2015; Chung et al. 2014; Chiu et al. 2012), while the other 4 studies did not describe the specific operation method (Cho et al. 2015; Song et al. 2015; Park et al. 2014; Choi et al. 2011). All of the studies were retrospective case–control studies, not randomized controlled trials (RCTs). The key characteristics of the studies are listed in Table 2.

**Table 2:** The key characteristics of the included studies

Year of publication	First author	Country	Duration	Patients	Mean age	Sex (M/F)	Depth of invasion (mucosal/submucosal)	Differentiation (differentiation/ undifferentiation)	Follow-up (month)	Score
2015	Cho JH	Korea	2003.5–2007.13	ESD 288 Surgery 173	ESD 62.2 ± 9.8 Surgery 59.4 ± 11.5	ESD 203/85 Surgery 115/58	ESD 268/20 Surgery 136/37	ESD 280/8 Surgery 104/69	ESD 77 Surgery 78	******
2015	Song WC	China	2007.8–2012.3	ESD 29 Surgery 59	ESD 65.3 ± 7.5 Surgery 45.8 ± 6.7	ESD 15/14 Surgery 38/21	ESD 27/2 Surgery 47/12	ESD 26/3 Surgery 47/12	ESD 26.9 ± 8.5 Surgery 22.3 ± 9.4	*****
2015	Choi IJ	Korea	2002.1–2007.12	ER 261 Surgery 114	ER 62 Surgery 62	ER 195/66 Surgery 88/26	NO	ER200/61 Surgery 75/39	ER74.9 Surgery 78.1	******
2015	Kim YI	Korea	2001–2009	ER 165 Surgery 292	ER 62 Surgery 60	ER 122/43 Surgery 217/75	ER 130/35 Surgery 250/42	ER156/9 Surgery 261/31	ER49.2 Surgery 59.3	*****
2014	Park CH	Korea	2007.1–2012.12	ESD 132 Surgery 132	ESD 73.9 ± 3.5 Surgery74.4 ± 3.7	ESD 97/35 Surgery 88/44	ESD123/9 Surgery 123/9	ESD 120/12 Surgery 121/11	ESD 17.6 Surgery 24.2	*****
2014	Kim DY	Korea	2004.1–2007.7	ESD 142 Surgery 71	ESD 62.0 ± 10.3 Surgery 56.7 ± 12.0	ESD 94/48 Surgery 58/13	ESD135/7 Surgery 62/9	ESD127/15 Surgery 54/17	ESD 76.7 ± 16.5 Surgery 65.5 ± 16.5	******
2014	Chung MW	Korea	2005.1–2010.12	ESD 76 Surgery 149	ESD 61.1 ± 12.6 Surgery 56.7 ± 12.8	ESD 44/32 Surgery 72/77	NO	NO	ESD 41.7 ± 22.6 Surgery 42.8 ± 17.3	*****
2012	Chiu PW	China	1993.1–2010.12	ESD 74 Surgery 40	ESD 66.3 Surgery 67	ESD 49/25 Surgery 23/17	ESD66/8 Surgery 19/21	NO	ESD 27 Surgery 77.6	*****
2011	Choi KS	Korea	1997.1–2002.8	EMR 172 Surgery 379	EMR 59.3 ± 9.1 Surgery 58.4 ± 10.3	EMR 127/45 Surgery 286/93	EMR 156/16 Surgery 342/37	NO	NO	******

### Local recurrence

**Fig. 2:**
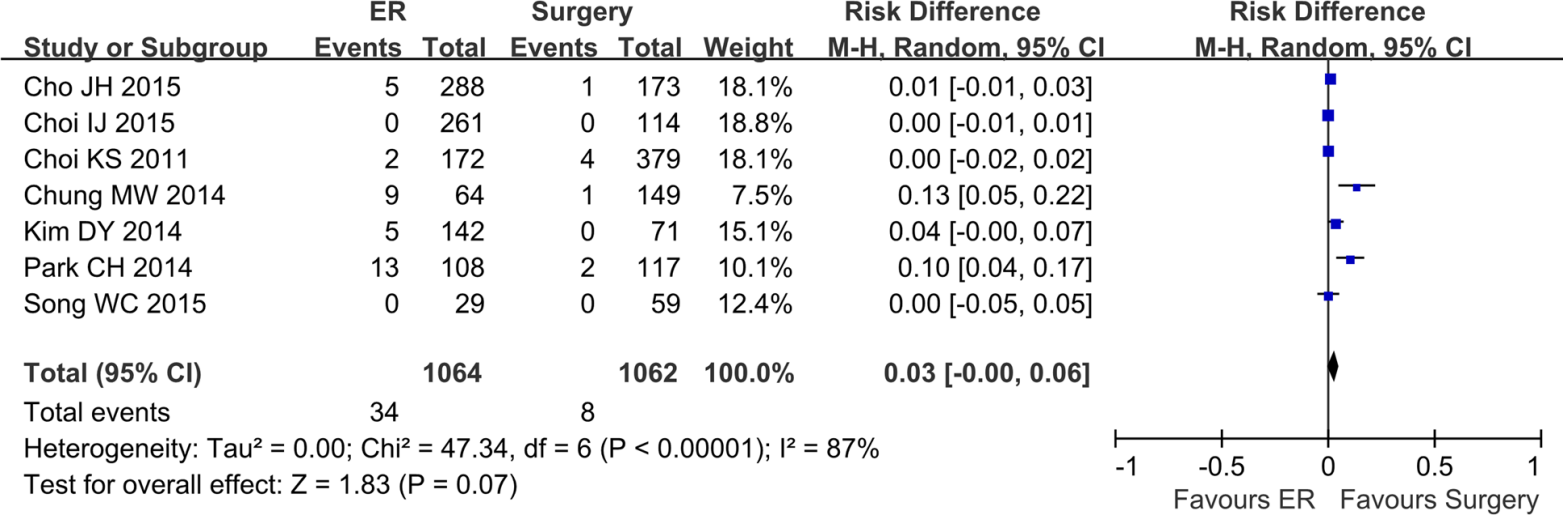
The rate of local recurrence comparing ER and surgery

The local recurrence rates were reported in 7 studies (Cho et al. 2015; Song et al. 2015; Choi et al. 2011, 2015; Park et al. 2014; Kim et al. 2014; Chung et al. 2014). A random effect model was applied because of the heterogeneity (*P* < 0.00001, I^2^ = 87 %). The analysis showed that the local recurrence rate was higher in the ER group (34/1064) than in the surgery group (8/1062) (OR 0.03; 95 % CI 0.00–0.06) (Fig. 2). When the study from China was excluded (Song et al. 2015), heterogeneity still existed (*P* < 0.00001, I^2^ = 90 %).

### Complications

**Fig. 3:**
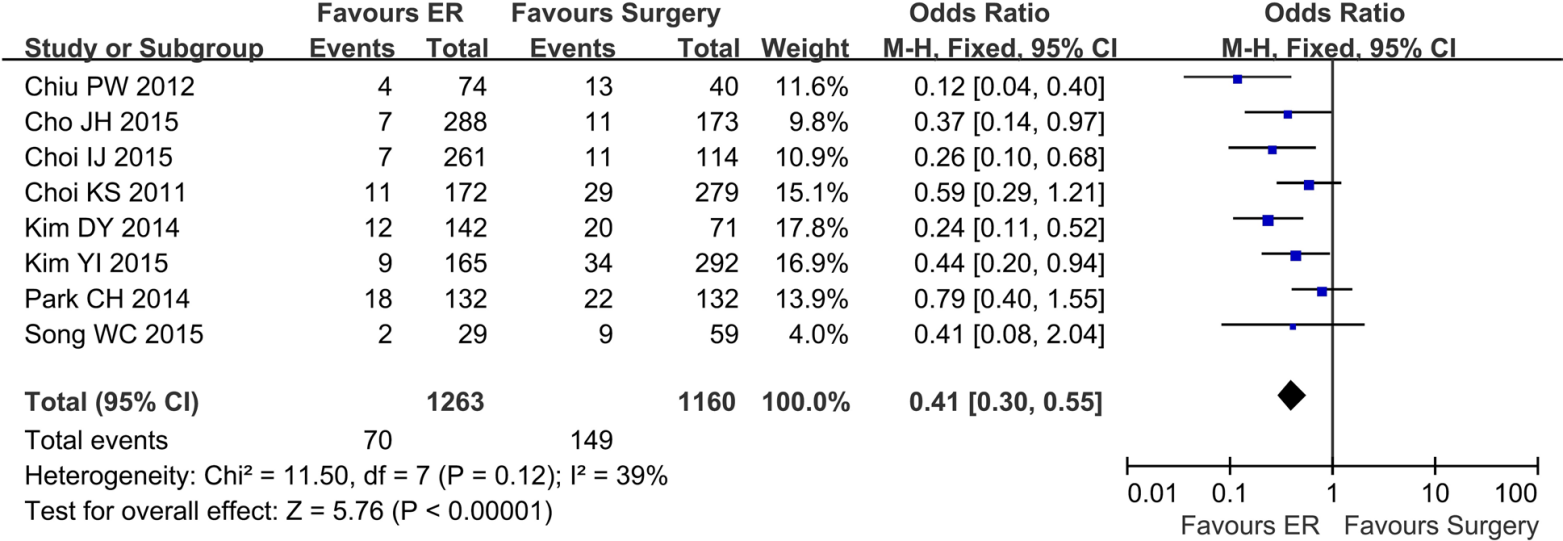
The rate of complications comparing ER and surgery

Complications were reported in 8 studies (Cho et al. 2015; Song et al. 2015; Choi et al. 2011, 2015; Kim et al. 2014, 2015; Park et al. 2014; Chiu et al. 2012). There was no heterogeneity among the studies (*P* = 0.12, I^2^ = 39 %). A fixed effect model was applied, and the subsequent analysis showed that the rate of complications was lower in the ER group (70/1263) than in the surgery group (149/1160) (OR 0.41; 95 % CI 0.30–0.55) (Fig. 3).

### Metachronous lesions

**Fig. 4:**
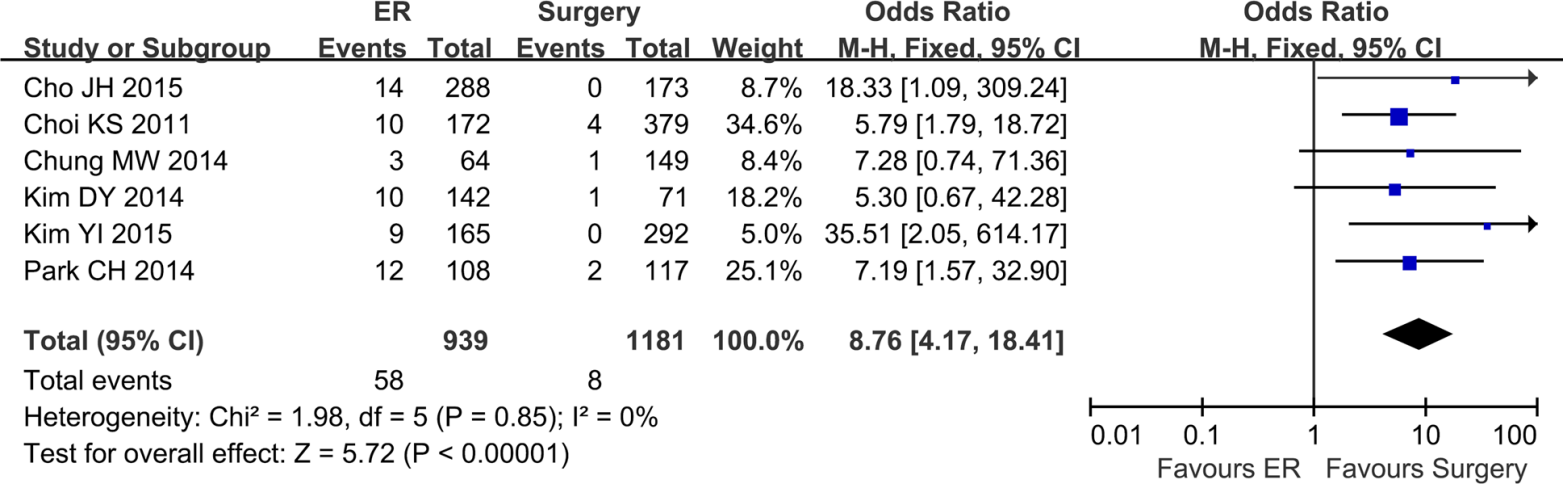
The rate of metachronous lesions comparing ER and surgery

Metachronous lesions were reported in 6 studies (Cho et al. 2015; Kim et al. 2014, 2015; Park et al. 2014; Chung et al. 2014; Choi et al. 2011). There was no heterogeneity in the studies (*P* = 0.85, I^2^ = 0 %), and a fixed effect model was applied. The rate of metachronous lesions was higher in the ER group (58/939) compared with the surgery group (8/1181) (OR 8.76; 95 % CI 4.17–18.41) (Fig. 4).

### Hospital stay

**Fig. 5:**
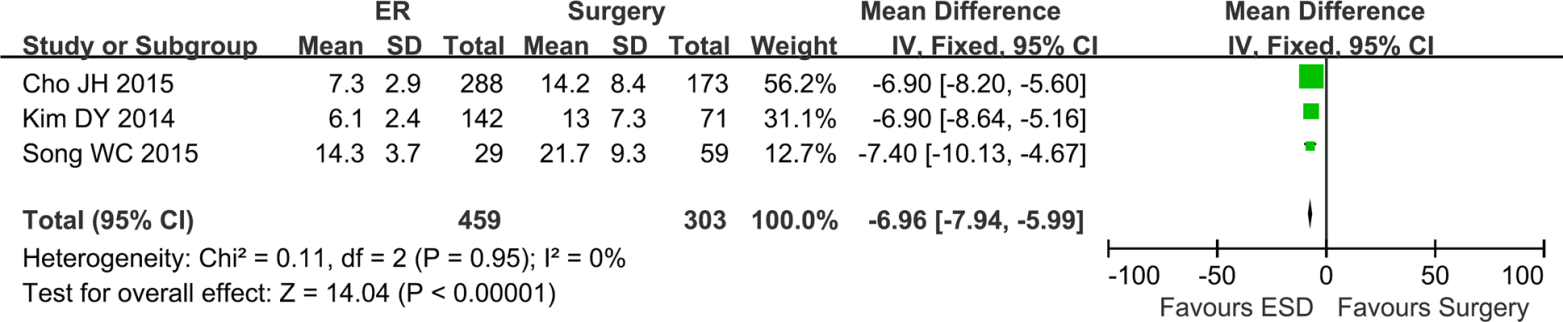
The hospital stay comparing ER and surgery

The hospital stay was reported in 3 studies (Cho et al. 2015; Song et al. 2015; Kim et al. 2014). There was no heterogeneity in the studies (*P* = 0.95, I^2^ = 0 %), and a fixed effect model was applied. The mean hospital stay was significantly shorter in the ER group, compared with the surgery group (Mean difference −6.96; 95 % CI −7.94 to −5.99) (Fig. 5).

### 5-Year overall survival

**Fig. 6:**
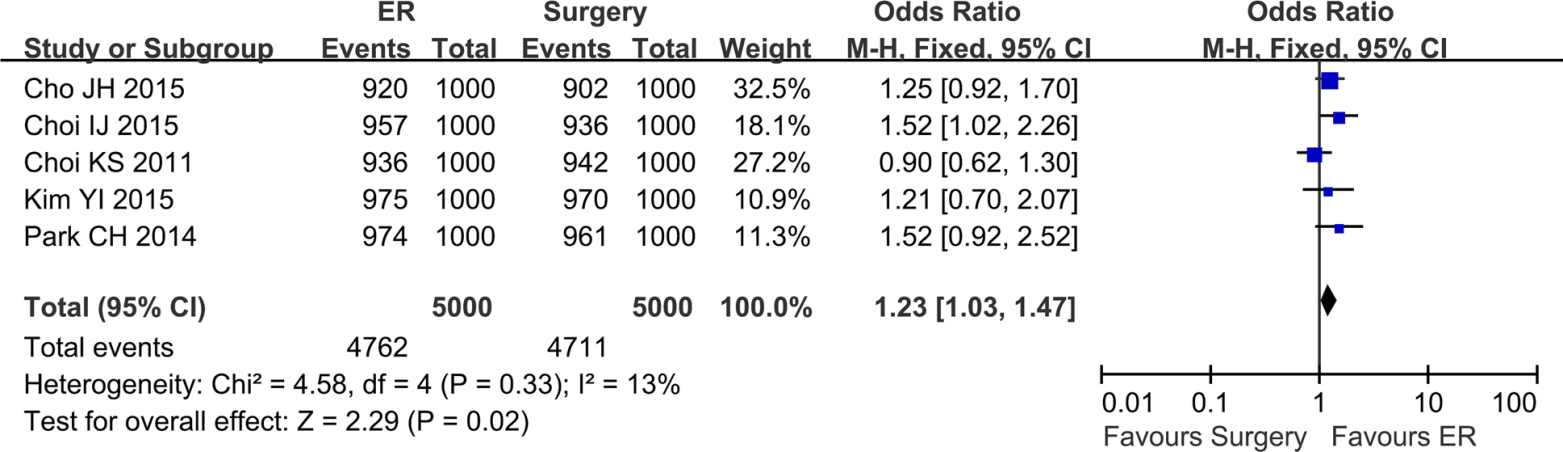
The 5-year overall survival comparing ER and surgery

The 5-year overall survival was reported in 5 studies (Cho et al. 2015; Song et al. 2015; Choi et al. 2011, 2015; Kim et al. 2015; Park et al. 2014). There was no heterogeneity in these studies (*P* = 0.33, I^2^ = 13 %), and a fixed effect model was applied. Analysis showed that there was no significant difference in the 5-year overall survival rate between the ER group and the surgery group (OR 1.23; 95 % CI 1.03–1.47) (Fig. 6).

### Publication bias

We used the local recurrence rate as the outcome, and no publication bias was detected by funnel plot and the Egger’s test (*P* = 0.862).

## Discussion

The comparison between ER and surgery in the treatment of early gastric cancer is still controversial. Even though ER is less invasive and less expensive, and could better preserve the physiological function of the stomach, additional surgery with lymph node dissection is recommended when ER is histologically non-curative or submucosal invasion is detected regardless of margin status because of possible residual tumors or lymph node metastasis (Oda et al. 2008; Song et al. 2008).

Therefore, we designed the meta-analysis to systematically evaluate the two techniques, providing evidence for the optimal treatment of early gastric cancer. In the present analysis, 9 retrospective studies were included, and the results confirmed that compared with surgery, ER had a higher rate of local recurrence and metachronous lesions for the treatment of early gastric cancer and precancerous lesions. Because the ER method preserves the whole stomach, the incidence of local recurrence and metachronous lesions after ER was higher than after surgery (Nakajima et al. 2006; Takeda et al. 1998; Hosokawa et al. 2002). In most cases, local recurrent and metachronous lesions were successfully cured with additional endoscopic treatments. That is why there was no significant difference in the 5-year overall survival rate between the two groups.

The complications of ER included bleeding and perforation, which can be managed using endoscopic treatment. The complications that occurred after gastrectomy included bleeding, duodenal leakage, ileus and hepatic dysfunction, and pancreatic leakage. 5 studies provided the data of the complication rate for EGC. The pooled analysis showed that it was higher in the surgery group than the ER group.

Five studies compared the hospital stay between the two groups, three studies provided the data of mean ± SD, and the mean hospital stay was significantly shorter in the ER group, compared with the surgery group.

Two studies (Choi et al. 2011; Kim et al. 2014) compared the medical cost between the two groups. The results showed that ER patients had lower medical costs than patients who had conventional surgeries for early gastric cancer. Choi et al. (2015) reported that endoscopic treatment for EGC provides a better quality of life, but stomach preservation might provoke cancer recurrence worries.

There is an alternative therapeutic approach for the treatment of early gastric cancer which combines the advantages of both surgical and endoscopic treatment also known as laparoscopic endoscopic cooperative surgery (LECS) which was introduced in 2009. It was reported that LECS held great promise for the future of minimally invasive oncologic procedures for the treatment of early gastric cancer (Ntourakis and Mavrogenis 2015). It can be used as an alternative to endoscopic therapy and surgical treatment.

There were certain limitations in our analysis. First, the major limitation of this meta-analysis was that none of the included studies were randomized. This certainly attenuated the evidence level and value of this meta-analysis. Second, included studies were from only 2 countries, Korea and China, so the results need further confirmation in other countries.

In conclusion, based on the findings of our meta-analysis, ER showed advantages over surgery for early gastric cancer and precancerous lesions regarding the procedure-related complication rate and hospital stay. The disadvantages of ER were the higher rate of local recurrence and metachronous lesions. Fortunately, we can treat these with additional endoscopic treatments without affecting overall survival. In the view of the present meta-analysis and all available trials, we suggest that ER is appropriate to most early gastric cancer and precancerous lesions.
